# Association of Pulmonary Tuberculosis and Diabetes in Mexico: Analysis of the National Tuberculosis Registry 2000–2012

**DOI:** 10.1371/journal.pone.0129312

**Published:** 2015-06-15

**Authors:** Guadalupe Delgado-Sánchez, Lourdes García-García, Martín Castellanos-Joya, Pablo Cruz-Hervert, Leticia Ferreyra-Reyes, Elizabeth Ferreira-Guerrero, Andrés Hernández, Victor Manuel Ortega-Baeza, Rogelio Montero-Campos, José Antonio Sulca, Ma. de Lourdes Martínez-Olivares, Norma Mongua-Rodríguez, Renata Baez-Saldaña, Jesús Felipe González-Roldán, Hugo López-Gatell, Alfredo Ponce-de-León, José Sifuentes-Osornio, María Eugenia Jiménez-Corona

**Affiliations:** 1 Centro de Investigación sobre Enfermedades Infecciosas, Instituto Nacional de Salud Pública, Cuernavaca, Morelos, México; 2 Dirección de Micobacteriosis, Centro Nacional de Programas Preventivos y Control de Enfermedades, México, Distrito Federal, México; 3 Departamento de Epidemiología, Instituto Nacional de Enfermedades Respiratorias, México, Distrito Federal, México; 4 Dirección General, Centro Nacional de Programas Preventivos y Control de Enfermedades, México, Distrito Federal, México; 5 Laboratorio de Microbiología, Instituto Nacional de Ciencias Médicas y de Nutrición “Salvador Zubirán”, México, Distrito Federal, México; 6 Dirección Médica, Instituto Nacional de Ciencias Médicas y de Nutrición “Salvador Zubirán”, México, Distrito Federal, México; 7 Dirección General Adjunta de Epidemiología, Dirección General de Epidemiología, México, Distrito Federal, México; University of California, Davis, UNITED STATES

## Abstract

**Background:**

Tuberculosis (TB) remains a public health problem in Mexico while the incidence of diabetes mellitus type 2 (DM) has increased rapidly in recent years.

**Objective:**

To describe the trends of incidence rates of pulmonary TB associated with DM and not associated with DM and to compare the results of treatment outcomes in patients with and without DM.

**Materials and Methods:**

We analysed the National Tuberculosis Registry from 2000 to 2012 including patients with pulmonary TB among individuals older than 20 years of age. The association between DM and treatment failure was analysed using logistic regression, accounting for clustering due to regional distribution.

**Results:**

In Mexico from 2000 to 2012, the incidence rates of pulmonary TB associated to DM increased by 82.64%, (p <0.001) in contrast to rates of pulmonary TB rate without DM, which decreased by 26.77%, (p <0.001). Patients with a prior diagnosis of DM had a greater likelihood of failing treatment (adjusted odds ratio, 1.34 (1.11–1.61) p <0.002) compared with patients who did not have DM. There was statistical evidence of interaction between DM and sex. The odds of treatment failure were increased in both sexes.

**Conclusion:**

Our data suggest that the growing DM epidemic has an impact on the rates of pulmonary TB. In addition, patients who suffer from both diseases have a greater probability of treatment failure.

## Introduction

Tuberculosis (TB) remains one of the main causes of morbidity and mortality in low- and medium-income countries, where the number of individuals with diabetes mellitus (DM) is rapidly increasing [1, 2]. For 2012, TB incidence rate in Mexico was of 23 per 100,000 inhabitants indicating that the disease continues to represent a public health problem; while DM prevalence of 9.17% among individuals older than 20 years of age ranks sixth among adults worldwide [1–3]. The convergence of both diseases in Mexico has led the International Diabetes Federation (IDF) to conclude that more than 10% of TB patients can be attributed to DM [4].

Many studies have explored the relationship between DM and TB, including a recent systematic review demonstrating that the risk of TB among people with DM triples that of people without DM [5]. Moreover, the available evidence indicates that DM comorbidity worsens the clinical outcomes of TB patients [6, 7].

Given the magnitude and clinical consequences of the association between DM and TB in Mexico, the objective of this study was to analyse incidence trends of TB and DM comorbidity and treatment outcomes according to DM during the period 2000 to 2012.

## Methodology

In Mexico, all TB patients are mandatorily reported according to official guidelines and registered in the National Tuberculosis Registry in Mexico [8]. Surveillance of TB was regulated by Mexican official norm and guidelines that were not modified during the study period. [8, 9] We analysed data from this registry including pulmonary TB patients aged 20 years or older who had been diagnosed during the years 2000 to 2012.

According to the official norms, TB patients were verified by acid fast bacilli (AFB) sputum smear, mycobacterial culture or histopathology [10]. Patients were treated under DOTS (directly observed treatment, short-course) strategy using the WHO (World Health Organization) standard regimen in which therapy was initiated with 4 drugs (2HRZE/4HR) for newly diagnosed patients and 5 drugs (2HRZES/1HRZE/5HRE) for previously treated patients all given under direct observation of treatment at the clinics. Patients harbouring isolates resistant to both isoniazid and rifampin were treated with a second-line standardised regime of at least 4 drugs that were highly likely to remain effective for 18–24 months after culture conversion [10].

Based on official guidelines, physicians diagnosed DM based on plasma glucose levels ≥120 mg/dl in fasting samples or ≥200 mg/dl in a 2 hour oral glucose tolerance test [11]. As defined in the Definitions section, for this study we used information on previous DM diagnosis self-referred by the patient. Patients with DM were treated according to official guidelines [11]. Briefly, treatment was based on early treatment goals, non-pharmacologic and pharmacologic treatment, patient education, self-monitoring and prevention of complications. Primary care physicians were encouraged to early use of combination therapy and timely addition of insulin in patients who did not achieve adequate glucose control. Limited resources of primary care centers many times limited the use HbA1c determination and therefore frequently treatment was based on venous and capillary glycemia. Primary care physicians also promoted adequate diet, weight control, physical activity, and community and family support.

### Mycobacteriology

Sputum samples were processed for acid fast bacilli smears and *M*. *tuberculosis* culture and drug susceptibility tests according to standardised procedures in the state laboratories [12]. The Institute of Diagnosis and Epidemiological Reference performed quality control analyses for all participating laboratories.

### Definitions

Following official surveillance guidelines [9], upon diagnosis of a TB case, health personnel mandatorily completed a case report form that standardized investigation of DM. The patient was considered to have DM if he/she self-referred to have been previously diagnosed by a physician. This question was asked using a standardized form in all the country throughout the study period. This definition may underestimate patients who are unaware of their diagnosis; however, it is used for epidemiological purposes by the Mexican health surveys and for surveillance purposes in other countries [13, 14].

The results of anti-tuberculosis treatment were defined according to official guidelines [10]. Briefly, failure was defined when AFB microscopies or cultures were positive at five months or later during treatment. Cure was defined when treatment was completed with the disappearance of signs and symptoms with two or more acid-fast bacilli smears or cultures with negative results at the end of therapy. Treatment completion was defined when a patient completed his/her treatment regimen with disappearance of signs and symptoms and smear or culture were not performed. Death was defined when a patient died of any cause during therapy. Treatment success was defined by the sum of patients who were cured and those who had completed treatment as defined above. Mexican guidelines have defined default when a patient interrupts treatment for 30 days or more rather than 60 days defined by WHO in order to be able to timely prevent that patients drop out from treatment.

We considered three regions, Mexico City and central Mexico, northern Mexico, and southern Mexico, according to regionalisation used in the National Survey of Health and Nutrition 2012 [15]. This regionalisation has been used by previous epidemiological studies to compare different areas in the country and is based on common geographic and socioeconomic characteristics: (Northern region: Baja California, Baja California Sur, Coahuila, Chihuahua, Durango, Nuevo León, Sonora, and Tamaulipas; Central Region: Aguascalientes, Colima, Guanajuato, Jalisco, México, Michoacán, Morelos, Nayarit, Querétaro, San Luis Potosí, Zacatecas, and Mexico City; and Southern Region: Campeche, Chiapas, Guerrero, Hidalgo, Oaxaca, Puebla, Quintana Roo, Tabasco, Tlaxcala, Veracruz, and Yucatán [16]. We combined information of Mexico City and Central Region given the geographical proximity and similar characteristics.

Following official guidelines for pulmonary TB among individuals 20 years or older, culture and antimicrobial susceptibility tests (AST) were performed only on patients who were suspected of harbouring drug resistant strains (subsequent treatments, persistence of acid fast bacilli (AFB) in sputum smear after fourth month of treatment, household contacts of drug resistant cases) [10, 17]. Isolates were tested for resistance to streptomycin, isoniazid, rifampicin and ethambutol. Antimicrobial susceptibility was determined according to three categories: 1) pansusceptible, if the isolate was susceptible to all drugs, 2) resistant, if it was resistant to at least one drug, excluding combined resistance to isoniazid and rifampin, and 3) multidrug resistant (MDR), if the isolate was resistant to isoniazid plus rifampin.

### Statistical analysis

Incidence rates were calculated using data from the TB registry as the numerator and census data developed by the National Institute of Geography and Informatics as denominator[18]. An annual population estimate was extrapolated for non-census years assuming a steady annual growth rate. We calculated percent change and performed the χ2 test for trends to detect significant annual trends overall and according to age group among patients with DM, without DM and overall.

Sociodemographic and clinical characteristics of patients with and without information on previous DM diagnosis were compared using Pearson's Chi or Kruskal-Wallis tests as appropriate.

Characteristics of patients according to diagnosis of DM, MDR TB and treatment failure were compared. Associations between sociodemographic and clinical characteristics with DM were tested by bivariate and multivariate logistic regression. Associations between DM and MDR TB and treatment failure were investigated by bivariate and multivariate logistic regression. All multivariate analyses accounted for clustering due to regional distribution. We tested for interaction between DM and sex. To assess whether sex modified the association of DM with the odds of MDR TB or treatment failure, we stratified by sex. Variables with p < 0.20 in the bivariate analysis and biological plausibility were included in multivariate models. We estimated the odds ratios (OR) and 95 per cent confidence intervals (CI), and identified the covariates that were independently associated with each outcome.

Given that only 1.26% (2,286/181,378) patients had AST results, we compared sociodemographic and clinical characteristics of patients with and without AST. The association between treatment failure and prior DM diagnosis was modelled using multivariate unconditional logistic regression as described above among patients with AST results.

All analyses were performed using the STATA 13.0 statistical software package (StataCorp LP, College Station, TX, USA).

### Ethical approval

Since this study was based on the retrospective analysis of the National Tuberculosis Registry, the “Comisión de Etica” of the Instituto Nacional de Salud Pública (approval number 422) approved the study and exempted the requirement of participants´ consent. Patient records/information was anonymized and de-identified prior to analysis.

## Results

During the study period, 191,923 patients were registered. Of these, information on previous DM diagnosis was available on 181,384 (94.51%). Comparison of patients with and without information of previous DM diagnosis is shown in S1 Table.

**Fig 1:**
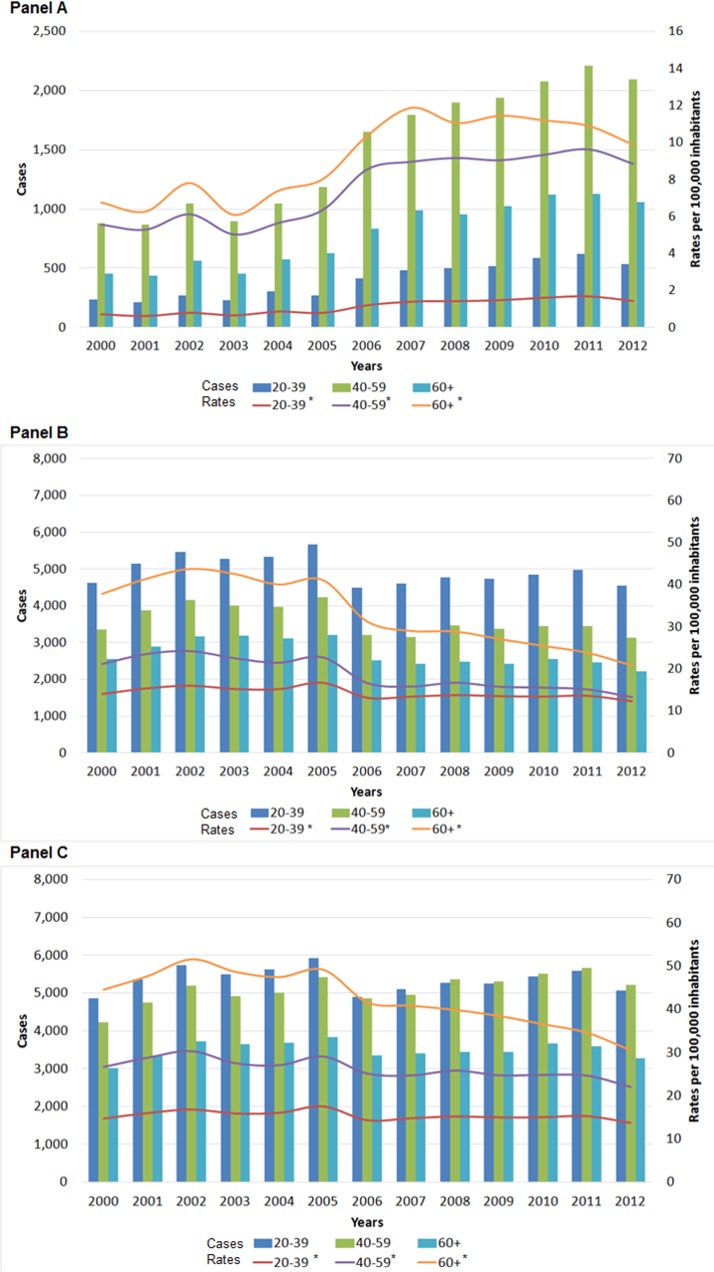
Trends of pulmonary TB rates and number of cases according to age groups and prior diagnosis of DM, Mexico 2000–2012. Number of pulmonary TB patients (bar) and incidence rates of pulmonary TB (line). Panel A: Pulmonary TB patients with a previous diagnosis of DM. Panel B: Pulmonary TB patients without a previous diagnosis of DM. Panel C: Total pulmonary TB patients with and without a previous diagnosis of DM. * p trend <0.001. TB; Tuberculosis; DM, Diabetes mellitus.

Of the 181,384 pulmonary TB patients, we excluded six individuals who did not have information on age. Of the 181,378 individuals who represented the study population, 19.29% (34,988) had been previously diagnosed with DM by a physician. During the study period, pulmonary TB with DM incidence rates increased by 82.64% (p trend <0.001) while rates of pulmonary TB without DM decreased by 26.77% (p trend<0.001) (Fig 1 and S2 Table).

Analysis of pulmonary TB associated with DM by age groups revealed incidence rates increased from 2000 to 2012 for all age groups (ptrend <0.001), (S2 Table). The highest rates were observed for the 60 and more and 40 to 59 year old groups. Incidence rates of pulmonary TB not associated with DM for all age groups decreased during the study period (ptrend <0.001). Highest rates occurred among the 60 and more year old individuals.

By bivariate analyses patients with TB and DM were more likely to be women, older, residents of Mexico City and Central region, have no access to social security, have been treated for a previous TB episode, have been diagnosed by AFB sputum smear, and harbour MDR strains. The proportion of patients who failed treatment was greater among patients with DM, but they experienced fewer deaths or treatment default (Table 1). Characteristics associated to DM by crude and adjusted analyses are shown in S3 Table. The adjusted models confirmed that age, sex, malnutrition, treatment for a previous TB episode (in patients without AST results) and drug resistance (patients with AST results) were all independently associated to DM.

**Table 1:** Characteristics of pulmonary TB patients according to DM diagnosis, Mexico 2000–2012.

Characteristic	Total	Pulmonary TB with DM	Pulmonary TB without DM	*p*-value*
	n = 181,378	n = 34,988 (19.29%)	n = 146,390 (80.370%)	
	Number/Total (%)	Number/Total (%)	Number/Total (%)	
Female	66,189/181,377 (36.49)	15,036/34,988 (42.97)	51,153/146,389 (34.94)	<0.001
Age (years) [median (IQR)]	46 (32–60)	52 (44–61)	43 (30–59)	<0.001**
Region				
Mexico City and Central region	45,963/181,371 (25.34)	9,214/34,987 (26.34)	36,749 / 146,384 (25.34)	<0.001†
Northern region	62,756/181,371 (34.60)	11,645/34,987 (33.38)	51,111/ 146,384 (34.60)	<0.001†
Southern region	72,652/181,371 (40.06)	14,128/34,987 (40.38)	58,524/ 146,384 (39.98)	0.169†
Lack of access to social security	51,646 /181,138 (28.51)	13,303/34,957 (38.06)	38,343/146,181 (26.23)	<0.001
Malnutrition	18,484/ 181,378 (10.19)	638/34,988 (1.82)	17,846/146,390 (12.19)	<0.001
Cirrhosis	351/181,378 (0.19)	49/ 34,988 (0.14)	302/146,390 (0.21)	0.011
Treatment for a previous TB episode	16,413 /178,780 (9.18)	3,338/34,413 (9.70)	13,075 /144,368 (9.06)	<0.001
Method for TB diagnosis				
Culture	1,935/181,092 (1.07)	373/34,966 (1.07)	1,562/146,126 (1.07)	0.972†
Sputum smear microscopy	153,050/181,092 (84.52)	30,962/34,966 (88.55)	122,088/146,126 (83.55)	<0.001†
Chest X rays	17,820 /181,092 (9.84)	2,365 /34,966 (6.76)	15,455/146,126 (10.58)	<0.001†
Histopathology	1,054 /181,092 (0.58)	155/34,966 (0.44)	899/146,126 (0.62)	<0.001†
Other	7,233/181,092 (3.99)	1,111/34,966 (3.18)	6,122/146,126 (4.19)	<0.001†
Antimicrobial susceptibility				
Pansusceptible	1,101/2,286 (48.16)	243/672 (36.16)	858/1,614 (53.16)	<0.001†
Resistant	1793/2,286 (8.44)	67/672 (9.97)	126/1,614 (7.81)	0.090†
MDR	992/2,286 (43.39)	362/672 (53.87)	630/1,614 (39.03)	<0.001†
Treatment outcome				
Cure and treatment completion	117,751/143,782 (81.90)	25,623/29,534 (86.76)	84,302/114,248 (80.54)	<0.001†
Failure	2,405/143,782 (1.67)	621/29,534 (2.10)	1,666/114,248 (1.59)	<0.001†
Default	11,188/143,782 (7.78)	1,317/29,534 (4.46)	9,043/114,248 (8.64)	<0.001†
Death during treatment	12,438/143,782 (8.65)	1,973 /29,534 (6.68)	10,465/114,248 (9.16)	<0.001†

TB, Tuberculosis; DM, Diabetes mellitus; OR, Odds Ratio; CI, Confidence Interval; IQR, Interquartile range; MDR, multidrug resistance.

* Chi-square test.

** Mann–Whitney Test.

† Binomial test.

We were interested in determining if DM was an independent risk factor for MDR TB. Characteristics of patients who harbor MDR strains compared to those of the rest of patients with antimicrobial susceptibility results and crude and adjusted analyses for variables associated to MDR TB are shown in S4 Table and S5 Table. When we examined modification of the association between DM and MDR by sex, we found statistically significant evidence of interaction (p = 0.027). We therefore performed a stratified analysis by sex. Among men, we found a significant association between DM and MDR (adjusted OR (aOR) 1.44 (1.17 to 1.76) but this association was not found among women (Table 2). These models were adjusted for sex (except in stratified by sex analysis), age, treatment for a previous TB episode, and malnutrition.

**Table 2:** Adjusted odds ratio (OR)* and 95% Confidence Intervals (CI) for MDR TB, Mexico 2000–2012.

Independent variable	No. of patients	Adjusted OR for MDR TB (95% CI)	*p*-value
All patients			
DM	1,874	1.28 (1.14 to 1.44)	<0.001
Women			
DM	553	1.06 (0.83 to 1.38)	0.604
Men			
DM	1,321	1.44 (1.17 to 1.76)	0.001

*Logistic regression analysis accounting for clustering due to regional distribution. All models were adjusted for sex (except in stratified by sex analysis), age, treatment for a previous TB episode, and malnutrition.

We analysed the association between DM and treatment failure. Characteristics of patients who failed treatment as compared to those of patients who were cured or completed treatment and crude and adjusted analyses for variables associated to treatment failure are shown in S6 Table and S7 Table. When we examined modification of the association between DM and treatment failure by sex, we found statistically significant evidence of interaction (p = 0.009). We therefore performed a stratified analysis by sex. In both women and men we found significant association aOR 1.62 (95% CI 1.31 to 2.01, *p*< 0.001) and aOR 1.21 (95% CI 1.01 to 1.44, *p* = 0.038), respectively. These models were adjusted for sex (except in stratified by sex analysis), age, treatment for a previous TB episode, year of diagnosis, and malnutrition, accounting for clustering due to regional distribution (Table 3).

**Table 3:** Adjusted odds ratio (OR)* and 95% Confidence Intervals (CI) for treatment failure, Mexico 2000–2012.

Independent variable	No. of patients	Adjusted OR for treatment failure (95%CI)	*p*-value
All patients			
DM	118,701	1.34 (1.11 to 1.61)	0.002
Women			
DM	45,754	1.62 (1.31 to 2.01)	<0.001
Men			
DM	72,947	1.21 (1.01 to 1.44)	0.038

DM, Diabetes mellitus; TB, Tuberculosis; OR, Odds Ratio; CI, Confidence Interval.

* Logistic regression analysis accounting for clustering due to regional distribution. All models were adjusted for sex (except in stratified by sex analysis), age, treatment for a previous TB episode, year of diagnosis, and malnutrition.

AST were performed in 1.26% (2,286/181,378) of patients´ isolates. These patients were more likely to be men, younger, with residence in the Northern region, to have access to social security; to have been previously diagnosed with DM or malnutrition, and to have been treated for a previous TB episode (S8 Table).

When we investigated the association between previous diagnoses of DM and treatment failure in the subgroup of patients who had information on AST, we did not find association between a previous diagnosis of DM and treatment failure. In this model, the highest risk of failure occurred among patients harbouring isoniazid and rifampin resistant isolates (OR 40.53, 95% CI 23.74 to 69.18, *p*< 0.001) compared with patients with pansusceptible isolates; the analyses were adjusted for previous diagnosis of DM, sex, age, previous TB treatment and malnutrition (Table 4).

**Table 4:** Association of treatment failure with DM and other patient characteristics among patients with pulmonary TB among the subgroup of patients with antimicrobial susceptibility results, by multivariate analyses* Mexico 2000–2012.

Variable	Adjusted OR (95% CI)	*p*-value
	n = 1,427	
DM	1.10 (0.72 to 1.66)	0.663
Female	0.72 (0.48 to 1.07)	0.105
Age (years)	0.99 (0.98 to 1.00)	0.140
Treatment for a previous TB episode	1.33 (0.92 to 1.92)	0.123
Malnutrition	3.22 (1.86 to 5.57)	<0.001
Antimicrobial susceptibility tests		
Pansusceptible	1.00	
Resistant	2.92 (1.23 to 6.95)	0.015
MDR	40.53 (23.74 to 69.18)	<0.001

DM, Diabetes mellitus; TB, Tuberculosis; MDR, multidrug resistant; OR, Odds Ratio; CI, Confidence Interval.

* Logistic regression analysis accounting for clustering due to regional distribution.

## Discussion

Analysis of pulmonary TB incidence rates in Mexico over the twelve years of our study revealed that while rates of TB without DM decreased, rates of TB and DM increased considerably. Results also suggest that TB pulmonary patients with a prior DM diagnosis were more likely to suffer treatment failure.

In Mexico, national health surveys have demonstrated that the prevalence of individuals with a prior diagnosis of DM has increased in recent years from 5.8% in 2000 to 7.3% in 2006 and to 9.17% in 2012 [3, 19]. In 2012, there were 6.4 million individuals with a diagnosis of DM, and this number could be even larger after taking into account the individuals who were unaware of their condition [3]. Given the emergence of DM and the endemicity of TB, we were interested in determining if the increasing rates of DM had impacted on TB rates. According to our data, the annual number of TB patients associated with DM increased by 134.20% (1,573 in 2000 to 3,684 in 2012), and the annual incidence rate increased by 82.64% (2.82 per 100,000 in 2000 to 5.16 per 100,000 in 2012), which represented 34,928 individuals over the study period. This information supports our prior findings in the southeast of Mexico, where we documented that 25% of pulmonary TB patients could be attributed to DM [20]. The frequency of DM among pulmonary TB patients observed in this study was high (19.29% %) and similar to what has been reported among Mexican Americans [21], and in other regions [22, 23]. The impact of DM on TB rates has been described in several studies. A systematic review revealed that the risk of TB among patients with DM triples that observed among patients who do not suffer from DM [5]. Using models that evaluated the interaction between different biological, environmental, and nutrition determinants, Dye and collaborators calculated that in India, the growing prevalence of DM (3.0% to 3.7%) increased the annual number of TB patients in individuals with DM by 46% between 1998 and 2008 and, increased the annual incidence rate by 24% [24]. Another study in India reported that a considerable proportion of new TB patients were attributed to DM (14.8% of pulmonary TB and 20.2% of sputum-positive TB, i.e., the transmissible form)[25]. The importance of TB incidence and its comorbidity with DM is highlighted by its association with 9.2% of the causes of death due to TB in the United States from 1990 to 2006. A total of 29.6% of the TB mortality due to DM occurred among Hispanic individuals [26].

The increase of pulmonary TB associated with DM rates occurred despite the successful implementation of the DOTs in Mexico [measured through case detection (75% in 2012)], a high proportion of treatment success (87% in 2012), and the near completion of the goals proposed by the WHO [1]. The explanation for this increase is complex and indicates that the control strategies implemented have not been totally efficacious because of the interaction of this disease with biological, environmental, and social determinants [24, 27]. Given the growing prevalence of DM in Mexico, it is likely that this chronic disease contributes to an increase in TB rates by increasing the host's susceptibility[28]. In addition, according to National Health Survey data from 2012, more than half of the individuals with DM (56.94%) came from low socioeconomic levels, and 16% did not have health insurance [3]. These data suggest that individuals who have DM and who live in poverty are exposed to previously known risks for TB, including a higher probability of being in contact with individuals who have active TB, a higher probability of living and/or working in overcrowded or ill ventilated conditions, a higher probability of food insecurity, less knowledge or empowerment regarding the need to adopt healthy habits (tobacco and alcohol use, diet), and limited access to good-quality health care services [29].

The increase of TB associated with DM rates occurred for all age groups during the study period. It is noticeable that TB associated with DM rates increased even among individuals younger than 40 years of age. This trend among younger individuals agrees with the increasing prevalence of DM among individuals 40 years old and younger (1.8% in 1993 to 5.8% in 2006) that has been observed in the National Health Surveys [19].

Although the physiopathology of TB susceptibility in patients with DM remains to be clarified, changes in the immune system have been described, including alterations in the complement pathway in patients with DM[30], increases in type 1 innate cytokines[31, 32], a reduction in the activation of alveolar macrophages [33], and increased IL-10 producing ability [34, 35]. Other authors have reported a reduction in Th1 cytokines[36]. Based on murine experiments, it has been suggested that TB susceptibility in DM can cause a delay in the initiation and expression of adaptive immunity [37]. In a recent review, the authors suggested that the interaction between the host and *M*. *tuberculosis* can be explained by the weakening of innate immunity followed by a hyper-reactive cell response [28].

Our data indicate that a prior diagnosis of DM is associated with treatment failure, which coincides with prior studies that have described negative outcomes among patients with TB and DM [6, 7]. The reason for this poor prognosis may be explained by abnormalities among patients with DM that affect immune response, including hyperglycaemia, microangiopathy, and alterations in renal function [28, 38, 39]. Furthermore, patients with DM have lower plasma concentrations of isoniazid and rifampin compared with patients without DM [40]. When we examined modification of the association between DM and treatment failure by sex, we found statistically significant evidence of interaction. When we stratified by sex, results showed increased adjusted odds for failure for both men and women. Sex and the other variables (age, previous treatment and malnutrition) that were included in our multivariate models have previously been associated with treatment failure [41–43].

Our finding that DM patients were more likely to harbour MDR strains supports previous findings, including a study conducted among Mexican residents of the border with the United States [44, 45]. Official Mexican guidelines recommend AST only on patients suspected to harbour drug resistant isolates. Selection for AST may have introduced a bias towards patients with different distribution of risk factors for treatment failure when compared with all pulmonary patients included in the Registry. We did not document association between previous diagnoses of DM and treatment failure among the subgroup of patients with AST, most probably because these patients differed in important variables including age, DM, and treatment for a previous TB episode from patients without AST as shown in S8 Table. This hypothesis is supported by a previous finding of our group where we conducted AST in all the study population and found that DM, previous treatment and MDR were independently associated to treatment failure [7].

### Study strengths

The study strengths include the following: 1) a population-based design that extended over a decade; 2) large study population; 3) among most patients (85.65%), diagnosis of TB was confirmed through sputum AFB smear or culture, and 4) the association between DM and treatment failure was adjusted for relevant variables.

### Study limitations

The limitations of this study are inherent to its design based on the National Tuberculosis Registry. We may have underestimated the real frequency of DM as the prevalence of DM was based on self-reporting of diagnosis by a physician. This method has been found to be adequate for epidemiological studies, although it underestimates the real frequency of DM by approximately 20% [14]. We did not have information on variables that would have been important to adjust for with regard to the association between DM and treatment outcome (*e*.*g*., blood glucose and glycosylated haemoglobin levels, time since DM diagnosis and medication). The study also does not reflect the long-term evolution of DM control as we did not measure metabolic control or complications. Finally, because information on antimicrobial susceptibility was available for only 1.23% of patients, we used information on prior treatment as a surrogate for AST in multivariable models.

### Conclusions

Our data indicate that the growing DM epidemic has impacted the frequency of pulmonary TB rates in Mexico. In addition, our data suggest that patients who suffer from both conditions exhibit a higher likelihood of therapeutic failure. These data indicate that DM prevention and control strategies should be integrated into TB control programs, and vice-versa, and their effectiveness should be carefully evaluated. The association between these diseases brings the risk of global dissemination and could have serious implications on the United Nations Millennium Goals compliance [25].

## Supporting Information

S1 Table Characteristics of pulmonary TB patients according to availability of information on prior diagnosis of DM, Mexico 2000–2012. (DOCX)

S2 Table Trends of pulmonary TB incidence rate according to prior diagnosis of DM and age group, Mexico 2000–2012. (DOCX)

S3 Table Characteristics associated to DM by crude and adjusted analyses, Mexico 2000–2012. (DOCX)

S4 Table Characteristics of pulmonary TB patients according to MDR TB, Mexico 2000–2012. (DOCX)

S5 Table Characteristics associated to MDR TB by crude and adjusted analyses, Mexico 2000–2012. (DOCX)

S6 Table Characteristics of pulmonary TB patients according to treatment failure, Mexico 2000–2012. (DOCX)

S7 Table Characteristics associated to treatment failure by crude and adjusted analyses, Mexico 2000–2012. (DOCX)

S8 Table Characteristics of pulmonary TB patients according to availability of antimicrobial susceptibility tests, Mexico 2000–2012. (DOCX)
