# Supplementary material for: Association of Pulmonary Tuberculosis and Diabetes in Mexico: Analysis of the National Tuberculosis Registry 2000–2012
Source: PLoS One. 2015 Jun 15;10(6):e0129312. doi: 10.1371/journal.pone.0129312 (PMC4468212; doi:10.1371/journal.pone.0129312)
Supplement: S1 Table — (DOCX) [file pone.0129312.s001.docx]

**S1 Table. Characteristics of pulmonary TB patients according to availability of information on prior diagnosis of DM, Mexico 2000-2012.**

| **Characteristic** | **Total** | **Without information on prior diagnosis of DM** | **With information on prior diagnosis of DM** | ***p*-value*** |
| --- | --- | --- | --- | --- |
|  | n= 191,923 | n= 10,539 (5.49%) | n= 181,384 (94.51%) |  |
|  | **Number/Total (%)** | **Number/Total (%)** | **Number/Total (%)** |  |
| Female | 70,195/191,916 (36.58) | 4,006/10,539 (38.01) | 66,189/181,377 (36.49) | 0.002 |
| Age (years) [median (IQR)] | 46 (32-60) | 45 (31-60) | 46 (32-59) | 0.002** |
| Region | | | | |
| Mexico City and Central region | 49,684 / 191,910 (25.89) | 3,721 / 10,539 (36.97) | 45,963 / 181,371 (25.89) | <0.001† |
| Northern region | 65,654 / 191,910 (34.21) | 2,898 / 10,539 (26.25) | 62,756 / 181,371 (34.21) | <0.001† |
| Southern region | 76,572 / 191,910 (39.90) | 3,241/ 10,539 (36.78) | 72,652 / 181,371 (39.90) | <0.001† |
| Lack of access to social security | 54,745/191,671 (28.56) | 3,099 / 10,533 (29.42) | 51,646/ 181,138 (28.51) | 0.044 |
| Treatment for a previous TB episode | 17,285 / 189,201 (9.14) | 872/10,421 (8.37) | 16,413/ 178,780 (9.18) | 0.005 |

* Chi-square test.; ** Mann–Whitney Test; † Binomial test. TB, Tuberculosis; DM, Diabetes mellitus; IQR, interquartile range.
