# Supplementary material for: Association of Pulmonary Tuberculosis and Diabetes in Mexico: Analysis of the National Tuberculosis Registry 2000–2012
Source: PLoS One. 2015 Jun 15;10(6):e0129312. doi: 10.1371/journal.pone.0129312 (PMC4468212; doi:10.1371/journal.pone.0129312)
Supplement: S2 Table — (DOCX) [file pone.0129312.s002.docx]

**S2 Table. Trends of pulmonary TB incidence rate* according to prior diagnosis of DM and age group, Mexico 2000-2012.**

| **Age group (years)** | | **Year** | | | | | | | | | | | | | | **2000-2012** | |
| --- | --- | --- | --- | --- | --- | --- | --- | --- | --- | --- | --- | --- | --- | --- | --- | --- | --- |
|  |  | **2000** | **2001** | **2002** | **2003** | **2004** | **2005** | **2006** | **2007** | **2008** | **2009** | **2010** | **2011** | **2012** | **Total** | **p trend** | **% of change** |
| **With DM** | | | | | | | | | | | | | | | | | |
| 20 to 39 | No. | 237 | 209 | 272 | 229 | 303 | 269 | 412 | 484 | 497 | 519 | 586 | 618 | 532 | 5,167 | <0.001 | 124.47 |
|  | Rate | 0.72 | 0.62 | 0.80 | 0.66 | 0.86 | 0.79 | 1.20 | 1.40 | 1.43 | 1.48 | 1.61 | 1.68 | 1.43 | 1.14 |  | 100.40 |
| 40 to 59 | No. | 880 | 871 | 1,049 | 895 | 1,046 | 1,183 | 1,652 | 1,795 | 1,901 | 1,941 | 2,077 | 2,209 | 2,093 | 19,592 | <0.001 | 137.84 |
|  | Rate | 5.55 | 5.28 | 6.11 | 5.02 | 5.65 | 6.35 | 8.55 | 8.96 | 9.16 | 9.04 | 9.34 | 9.63 | 8.85 | 7.69 |  | 59.50 |
| 60 + | No. | 456 | 438 | 565 | 456 | 574 | 625 | 835 | 990 | 954 | 1,023 | 1,124 | 1,130 | 1,059 | 10,229 | <0.001 | 132.24 |
|  | Rate | 6.75 | 6.27 | 7.81 | 6.09 | 7.40 | 8.03 | 10.38 | 11.89 | 11.06 | 11.45 | 11.20 | 10.90 | 9.88 | 9.38 |  | 46.31 |
| All | No. | 1,573 | 1,518 | 1,886 | 1,580 | 1,923 | 2,077 | 2,899 | 3,269 | 3,352 | 3,483 | 3,787 | 3,957 | 3,684 | 34,928 | <0.001 | 134.20 |
|  | Rate | 2.82 | 2.66 | 3.22 | 2.63 | 3.13 | 3.44 | 4.61 | 5.19 | 5.22 | 5.32 | 5.52 | 5.65 | 5.16 | 4.27 |  | 82.64 |
| **Without DM** | | | | | | | | | | | | | | | | | |
| 20 to 39 | No. | 4,623 | 5,152 | 5,458 | 5,268 | 5,326 | 5,661 | 4,482 | 4,611 | 4,776 | 4,726 | 4,849 | 4,975 | 4,537 | 64,444 | <0.001 | -1.86 |
|  | Rate | 13.96 | 15.30 | 15.96 | 15.17 | 15.13 | 16.66 | 13.08 | 13.34 | 13.70 | 13.45 | 13.34 | 13.55 | 12.23 | 14.20 |  | -12.38 |
| 40 to 59 | No. | 3,347 | 3,877 | 4,148 | 4,012 | 3,965 | 4,229 | 3,207 | 3,151 | 3,458 | 3,369 | 3,440 | 3,453 | 3,126 | 46,782 | <0.001 | -6.60 |
|  | Rate | 21.11 | 23.50 | 24.18 | 22.50 | 21.41 | 22.71 | 16.60 | 15.73 | 16.67 | 15.70 | 15.48 | 15.05 | 13.22 | 18.36 |  | -37.37 |
| 60 + | No. | 2,553 | 2,894 | 3,166 | 3,190 | 3,109 | 3,201 | 2,514 | 2,412 | 2,485 | 2,415 | 2,547 | 2,460 | 2,218 | 35,164 | <0.001 | -13.12 |
|  | Rate | 37.81 | 41.41 | 43.75 | 42.57 | 40.06 | 41.12 | 31.24 | 28.96 | 28.82 | 27.04 | 25.38 | 23.73 | 20.70 | 32.24 |  | -45.27 |
| All | No. | 10,523 | 11,923 | 12,772 | 12,470 | 12,400 | 13,091 | 10,203 | 10,174 | 10,719 | 10,510 | 10,836 | 10,888 | 9,881 | 146,390 | <0.001 | -6.10 |
|  | Rate | 18.89 | 20.86 | 21.80 | 20.77 | 20.17 | 21.68 | 16.56 | 16.17 | 16.69 | 16.04 | 15.79 | 15.55 | 13.83 | 17.90 |  | -26.77 |
| **All** | | | | | | | | | | | | | | | | | |
| 20 to 39 | No. | 4,860 | 5,361 | 5,730 | 5,497 | 5,629 | 5,930 | 4,894 | 5,095 | 5,273 | 5,245 | 5,435 | 5,593 | 5,069 | 69,611 | <0.001 | 4.30 |
|  | Rate | 14.68 | 15.92 | 16.75 | 15.83 | 15.99 | 17.46 | 14.28 | 14.74 | 15.12 | 14.93 | 14.95 | 15.23 | 13.67 | 15.34 |  | -6.88 |
| 40 to 59 | No. | 4,227 | 4,748 | 5,197 | 4,907 | 5,011 | 5,412 | 4,859 | 4,946 | 5,359 | 5,310 | 5,517 | 5,662 | 5,219 | 66,374 | <0.001 | 23.47 |
|  | Rate | 26.66 | 28.78 | 30.29 | 27.52 | 27.06 | 29.06 | 25.15 | 24.70 | 25.84 | 24.74 | 24.82 | 24.68 | 22.07 | 26.04 |  | -17.20 |
| 60 + | No. | 3,009 | 3,332 | 3,731 | 3,646 | 3,683 | 3,826 | 3,349 | 3,402 | 3,439 | 3,438 | 3,671 | 3,590 | 3,277 | 45,393 | <0.001 | 8.91 |
|  | Rate | 44.56 | 47.67 | 51.56 | 48.66 | 47.45 | 49.15 | 41.61 | 40.84 | 39.88 | 38.49 | 36.58 | 34.63 | 30.58 | 41.62 |  | -31.39 |
| All | No. | 12,096 | 13,441 | 14,658 | 14,050 | 14,323 | 15,168 | 13,102 | 13,443 | 14,071 | 13,993 | 14,623 | 14,845 | 13,565 | 181,378 | <0.001 | 12.14 |
|  | Rate | 21.71 | 23.52 | 25.01 | 23.40 | 23.29 | 25.12 | 21.26 | 21.36 | 21.91 | 21.36 | 21.31 | 21.20 | 18.99 | 22.18 |  | -12.54 |

* Rate per 100,000 inhabitants. TB, Tuberculosis; DM, Diabetes mellitus.
