# Supplementary material for: Association of Pulmonary Tuberculosis and Diabetes in Mexico: Analysis of the National Tuberculosis Registry 2000–2012
Source: PLoS One. 2015 Jun 15;10(6):e0129312. doi: 10.1371/journal.pone.0129312 (PMC4468212; doi:10.1371/journal.pone.0129312)
Supplement: S3 Table — (DOCX) [file pone.0129312.s003.docx]

**S3 Table. Characteristics associated to DM by crude and adjusted* analyses. Mexico 2000-2012.**

| Characteristic | Total | Crude OR (95% CI) | Adjusted OR (95% CI)** | Adjusted OR*(95% CI)*** |
| --- | --- | --- | --- | --- |
|  |  |  | n=178,772 | n=1,874 |
|  | Number/Total (%) |  |  |  |
| Female | 66,189/181,377 (36.49) | 1.40 (1.37 to 1.44) | 1.44 (1.23 to 1.68) | 2.14 (1.75 to 2.63) |
| Age |  |  |  |  |
| 20 to 39 years | 69,611/181,378 (38.38) | 1.00 | 1.00 | 1.00 |
| 40 to 59 years | 5,167/181,378 (36.59) | 2.71 (2.65 to 23.77) | 5.18 (4.74 to 5.66) | 5.91 (4.63 to 7.54) |
| 60 years and more | 45,393181,378 (25.03) | 1.31 (1.27 to 1.34) | 3.66 (3.01 to 4.44) | 4.13 (2.43 to 7.01) |
| Region | | | | |
| Mexico City and Central region | 45,963/181,371 (25.34) | 1.00 | --- | --- |
| Northern region | 62,756/181,371 (34.60) | 0.93 (0.91 to 0.95) | --- | --- |
| Southern region | 72,652/181,371 (40.06) | 1.01 (0.99 to 1.04) | --- | --- |
| Lack of access to social security | 51,646 /181,138 (28.51) | 1.73 (1.69 to 1.77) | --- | --- |
| Malnutrition | 18,484/ 181,378 (10.19) | 0.13 (0.12 to 0.15) | 0.13 (0.07 to 0.24) | 0.010 (0.074 to 0.14) |
| Cirrhosis | 351/181,378 (0.19) | 0.68 (0.49 to 0.92) | --- | --- |
| Treatment for a previous TB episode | 16,413 /178,780 (9.18) | 1.08 (1.04 to 1.12) | 1.17 (1.06 to 1.29) | 1.14 (0.92 to 1.40) |
| Antimicrobial susceptibility tests |  |  |  |  |
| Pansusceptible | 1,101/2,286 (48.16) | 1.00 | --- | 1.00 |
| Resistant | 1793/2,286 (8.44) | 1.31 (0.94 to 1.80) | --- | 2.03 (1.13 to 3.66) |
| MDR | 992/2,286 (43.39) | 1.82 (1.51 to 2.20) | --- | 1.45 (1.18 to 1.78) |

* Logistic regression analysis accounting for clustering due to regional distribution; ** Patients without antimicrobial susceptibility tests; *** Patients with antimicrobial susceptibility tests. DM, Diabetes mellitus, TB, Tuberculosis; OR, Odds Ratio; CI, Confidence Interval; MDR, multidrug resistance.
