# Supplementary material for: Association of Pulmonary Tuberculosis and Diabetes in Mexico: Analysis of the National Tuberculosis Registry 2000–2012
Source: PLoS One. 2015 Jun 15;10(6):e0129312. doi: 10.1371/journal.pone.0129312 (PMC4468212; doi:10.1371/journal.pone.0129312)
Supplement: S4 Table — (DOCX) [file pone.0129312.s004.docx]

**S4 Table. Characteristics of pulmonary TB patients according to MDR, Mexico 2000-2012.**

| Characteristic | Total  n= 2,286 | Pulmonary TB with MDR  n= 992 (43.40%) | Pulmonary TB without MDR  n= 1,294 (56.60%) | p-value*,** |
| --- | --- | --- | --- | --- |
|  | Number/Total (%) | Number/Total (%) | Number/Total (%) |  |
| DM | 672/2,286 (29.40) | 362/992 (36.49) | 310/1,294 (23.96) | <0.001 |
| Female | 680/2,286 (29.75) | 309/992 (31.15) | 37/1,294 (28.67) | 0.199 |
| Age (years) [median (IQR)] | 43 (31-54) | 43 (32-54) | 43 (31-55) | 0.838*** |
| Region | | | | |
| Mexico City and Central region | 596/2,447 (24.36) | 235/1,070 (21.96) | 361/1,377 (26.22) | 0.003† |
| Northern region | 1,316/2,447 (53.78) | 547/1,070 (51.12) | 769/1,377 (55.85) | 0.023† |
| Southern region | 535/2,447 (21.86) | 288/1,070 (26.92) | 247/1,377 (17.94) | <0.001† |
| Lack of access to social security | 339 /2278 (14.88) | 192/988 (19.43) | 147/1,290 (11.40) | <0.001 |
| Malnutrition | 304/2,286 (13.30) | 122/992 (12.30) | 182/1,294 (14.06) | 0.218 |
| Cirrhosis | 2/2,286 (0.09) | 2/992 (0.20) | 0/1,294 (0.00) | 0.106 |
| Treatment for a previous TB episode | 642 /1,875 (34.24) | 292/628 (46.50) | 350 /1,247 (28.07) | <0.001 |

*MDR versus pansusceptible and resistant; ** Chi-square test; ***Mann–Whitney Test; †Binomial test. TB, Tuberculosis; DM, diabetes mellitus; IQR, interquartile range; MDR, multidrug resistance.
