# Supplementary material for: Association of Pulmonary Tuberculosis and Diabetes in Mexico: Analysis of the National Tuberculosis Registry 2000–2012
Source: PLoS One. 2015 Jun 15;10(6):e0129312. doi: 10.1371/journal.pone.0129312 (PMC4468212; doi:10.1371/journal.pone.0129312)
Supplement: S5 Table — (DOCX) [file pone.0129312.s005.docx]

**S5 Table. Characteristics associated to MDR TB by crude and adjusted analyses. Mexico 2000-2012.**

| Characteristic | Crude analysis | | Adjusted* analysis | |
| --- | --- | --- | --- | --- |
|  | OR (95% CI) | p-value | OR (95% CI) | p-value |
|  | n = 2,286 |  | n = 1,874 |  |
| DM | 1.82 (1.52 to 2.20) | <0.001 | 1.28 (1.14 to 1.44) | <0.001 |
| Female | 1.13 (0.94 to 1.35) | 0.199 | 1.11 (1.06 to 1.16) | <0.001 |
| Age (years) |  | | | |
| 20 to 39 | 1.00 |  | 1.00 |  |
| 40 to 59 | 1.18 (0.99 to 1.40) | 0.058 | 0.97 (0.80 to 1.17) | 0.746 |
| 60 and more | 0.79 (0.62 to 0.99) | 0.042 | 0.79 (0.56 to 1.11) | 0.169 |
| Treatment for a previous TB episode | 2.23 (1.81 to 2.73) | <0.001 | 2.28 (1.10 to 4.72) | 0.027 |
| Malnutrition | 0.86 (0.66 to 1.10) | 0.218 | 0.72 (0.56 to 1.13) | 0.151 |

* Logistic regression analysis accounting for clustering due to regional distribution; DM, Diabetes mellitus; MDR, multidrug resistance; TB, Tuberculosis; OR, Odds Ratio; CI, Confidence Interval.
