# Supplementary material for: Association of Pulmonary Tuberculosis and Diabetes in Mexico: Analysis of the National Tuberculosis Registry 2000–2012
Source: PLoS One. 2015 Jun 15;10(6):e0129312. doi: 10.1371/journal.pone.0129312 (PMC4468212; doi:10.1371/journal.pone.0129312)
Supplement: S6 Table — (DOCX) [file pone.0129312.s006.docx]

**S6 Table. Characteristics of pulmonary TB patients according to treatment failure. Mexico 2000-2012.**

| Characteristic | Total | Pulmonary TB who failed treatment | Pulmonary TB patients who cured or completed treatment | p-value*,** |
| --- | --- | --- | --- | --- |
|  | n= 120,155 | n= 2,405 (2.00%) | n= 117,750 (98.00%) |  |
|  | Number/Total (%) | Number/Total (%) | Number/Total (%) |  |
| Female | 46,265/120,155 (38.5) | 760/2,405 (31.60) | 45,505/117,750 (38.65) | <0.001 |
| Age (years) [median (IQR)] | 46 (33-59) | 45 (34-57) | 46 (32-59) | 0.164*** |
| Region | | | | |
| Mexico City and Central region | 31,823/120,155 (26.48) | 422/2,405 (17.55) | 31,401/117,750 (26.67) | <0.001† |
| Northern region | 38,785/120,155 (32.28) | 906/2,405 (37.67) | 37,869/117,750 (32.17) | <0.001† |
| Southern region | 49,597/120,155 (41.24) | 1,077/2,405 (44.78) | 48,470/117,750 (41.16) | <0.001† |
| Lack of access to social security | 34,345/120,001 (28.62) | 448/2,395 (18.75) | 33,897/117,606 (28.82) | <0.001 |
| DM | 26,244/120,156 (21.84) | 621/2,45 (25.82) | 25,623/117,751 (21.76) | <0.001 |
| Malnutrition | 12,545/120,156 (10.44) | 326/2,405 (13.56) | 12,219/117,751 (10.38) | <0.001 |
| Cirrhosis | 186/120,156 (0.15) | 7/2,405 (0.29) | 179/117,751 (0.15) | 0.086 |
| Treatment for a previous TB episode | 9,258/18,703 (7.80) | 694/2,283 (30.40) | 8,564/116,420 (7.29) | <0.001 |
| Method for TB diagnosis | | | | |
| Culture | 1,118/120,083 (0.93) | 61/2,404 (2.54) | 1,057/117,679 (0.90) | <0.001† |
| Sputum smear microscopy | 101,724/120,083 (86.55) | 2,213/2,404 (92.05) | 101,724/117,679 (86.44) | <0.001† |
| Chest X rays | 10,104/120,083 (8.49) | 91/2,404 (3.79) | 10,104/117,679 (8.59) | <0.001† |
| Histopathology | 659/120,083 (0.55) | 3/2,404 (0.12) | 659/117,679 (0.56) | 0.004 |
| Other | 4,135/120,083 (3.47) | 36/2,404 (1.55) | 4,135/117,679 (3.51) | <0.001† |
| Antimicrobial susceptibility tests | | | | |
| Pansusceptible | 874/1,604 (54.49) | 21/244 (8.61) | 853/1,360 (62.72) | <0.001† |
| Resistant | 156/1,604 (9.73) | 12/244 (4.92) | 144/1,360 (10.59) | 0.090† |
| MDR | 574/1,604 (35.79) | 211/244 (86.48) | 363/1,360 (26.69) | <0.001† |

* Treatment failure versus cure and treatment completion; ** Chi-square test; ***Mann–Whitney Test; †Binomial test. TB, Tuberculosis; DM, diabetes mellitus; IQR, interquartile range; MDR, multidrug resistance.
