# Supplementary material for: Association of Pulmonary Tuberculosis and Diabetes in Mexico: Analysis of the National Tuberculosis Registry 2000–2012
Source: PLoS One. 2015 Jun 15;10(6):e0129312. doi: 10.1371/journal.pone.0129312 (PMC4468212; doi:10.1371/journal.pone.0129312)
Supplement: S7 Table — (DOCX) [file pone.0129312.s007.docx]

**S7 Table. Characteristics associated to treatment failure by crude and adjusted analyses. Mexico 2000-2012.**

| Characteristic | Crude OR (95% CI) | | Adjusted* OR (95% CI) | |
| --- | --- | --- | --- | --- |
|  | n = 120,155 | p-value | n = 118,701 | p-value |
| DM | 1.25 (1.14 to 1.37) | <0.001 | 1.34 (1.11 to 1.61) | 0.002 |
| Female | 0.73 (0.67 to 0.80) | <0.001 | 0.77 (0.70 to 0.83) | <0.001 |
| Age (years) |  | | | |
| 20 to 39 | 1.00 |  | 1.00 |  |
| 40 to 59 | 1.16 (1.07 to 1.26) | <0.001 | 1.01 (0.92 to 1.11) | 0.798 |
| 60 and more | 0.81 (0.74 to 0.90) | <0.001 | 0.86 (0.085 to 0.86) | <0.001 |
| Treatment for a previous TB episode | 5.50 (5.01 to 6.04) | <0.001 | 5.34 (4.65 to 6.15) | <0.001 |
| Malnutrition | 1.35 (1.20 to 1.53) | <0.001 | 1.32 (1.17 to 1.48) | <0.001 |
| Year of diagnosis | | | | |
| 2000 | 1.00 |  | 1.00 |  |
| 2001 | 1.08 (0.87 to 1.35) | 0.430 | 1.27 ( 1.17 to 1.37) | <0.001 |
| 2002 | 1.09 (0.94 to 1.25) | 0.240 | 1.15 (1.06 to 1.24) | 0.001 |
| 2003 | 1.05 (0.87 to 1.26) | 0.620 | 1.14 (1.02 to 1.29) | 0.026 |
| 2004 | 0.81 (0.65 to 0.99) | 0.048 | 0.96 (0.81 to 1.12) | 0.578 |
| 2005 | 0.81 (0.66 to 0.99) | 0.034 | 0.92 (0.71 to 1.20) | 0.550 |
| 2006 | 0.87 (0.75 to 1.02) | 0.078 | 0.87 (0.70 to 1.08) | 0.212 |
| 2007 | 1.06 (0.92 to 1.22) | 0.387 | 0.95 (0.77 to 1.15) | 0.591 |
| 2008 | 0.95 (0.82 to 1.09) | 0.461 | 0.91 (0.76 to 1.09) | 0.299 |
| 2009 | 1.01 (0.89 to 1.17) | 0.790 | 0.93 (0.81 to 1.07) | 0.344 |
| 2010 | 1.06 (0.93 to 1.21) | 0.358 | 1.04 (1.02 to 1.08) | 0.002 |
| 2011 | 1.03 (0.90 to 1.18) | 0.653 | 1.02 (0.91 to 1.13) | 0.775 |
| 2012 | 1.14 (0.98 to 1.32) | 0.076 | 1.16 (0.97 to 1.38) | 0.094 |

* Logistic regression analysis accounting for clustering due to regional distribution. DM, Diabetes mellitus; OR, Odds Ratio; CI, Confidence Interval; TB, Tuberculosis.
