# Supplementary material for: Association of Pulmonary Tuberculosis and Diabetes in Mexico: Analysis of the National Tuberculosis Registry 2000–2012
Source: PLoS One. 2015 Jun 15;10(6):e0129312. doi: 10.1371/journal.pone.0129312 (PMC4468212; doi:10.1371/journal.pone.0129312)
Supplement: S8 Table — (DOCX) [file pone.0129312.s008.docx]

**S8 Table. Characteristics of pulmonary TB patients according to availability of antimicrobial susceptibility tests, Mexico 2000-2012.**

| Characteristic | Total | With information on antimicrobial susceptibility tests | Without information on antimicrobial susceptibility tests | p-value* |
| --- | --- | --- | --- | --- |
|  | Number/Total (%) | Number/Total (%) | Number/Total (%) |  |
|  | n= 181,378 | n= 2,286 (1.26%) | n= 179,092 (98.74%) |  |
| Female | 66,189/181,377 (36.49) | 680/2,286 (29.75) | 65,509 /179,091 (36.58) | <0.001 |
| Age (years) [median (IQR)] | 46 (32-60) | 43 (31-54) | 46 (32-60) | <0.001** |
| Region | | | | |
| Mexico City and Central region | 45,963/181,371 (25.37) | 531/2,285 (23.24) | 45,432/179,086 (25.34) | 0.020† |
| Northern region | 62,756/181,371 (34.35) | 1,241/2,285 (54.31) | 61,515/179,086 (34.60) | <0.001† |
| Southern region | 72,652/181,371 (40.28) | 513 /2,285 (22.45) | 72,139/179,086 (40.06) | <0.001† |
| Lack of access to social security | 51,646/181,138 (28.51) | 339/2,078 (14.88) | 51,307/178,860 (28.69) | <0.001 |
| Treatment for a previous TB episode | 16,413/178,780 (9.18) | 642/1,875 (34.24) | 15,771/176,905 (8.91) | <0.001 |
| Previous diagnoses of DM | 34,988/181,378 (19.29) | 672/2,286 (29.40) | 34,616/179,092 (19.16) | <0.001 |
| Previous diagnoses of malnutrition | 18,484/181,378 (10.19) | 304/2,286 (13.30) | 18,180/179,092 (10.15) | <0.001 |
| Previous diagnoses of cirrhosis | 35/181,378 (0.19) | 2/2,286 (0.09) | 349/179,092 (0.19) | 0.246 |

* Chi-square test; ** Mann–Whitney Test; † Binomial test; TB, Tuberculosis; DM, Diabetes mellitus; IQR, interquartile range.
